# Motor Function Profiling and Its Impact on Health-Related Quality of Life in Childhood Stroke Survivors

**DOI:** 10.1016/j.arrct.2025.100578

**Published:** 2025-12-19

**Authors:** Chai Yin Charlie Fan, Yuliang Wang, Wan Yee Winnie Tso

**Affiliations:** a Department of Paediatrics and Adolescent Medicine, The University of Hong Kong, Pokfulam, Hong Kong SAR, China; b Department of Psychology, The University of Hong Kong, Pokfulam, Hong Kong SAR, China; c The Duchess of Kent Children’s Hospital, Hong Kong SAR, China; d The Hong Kong Children’s Hospital, Hong Kong SAR, China

**Keywords:** Childhood stroke, Outcome, Motor, Quality of life, Psychosocial, Rehabilitation

## Abstract

**Objective:**

To profile motor function in childhood stroke survivors and examine the association between motor deficits and health-related quality of life (HRQoL) in this population.

**Design:**

Cross-sectional.

**Setting:**

Pediatric neurology and rehabilitation clinic.

**Participants:**

Twenty-four childhood stroke survivors (N=24) (9 girls; mean age at assessment=13.3±3.5y).

**Interventions:**

Not applicable.

**Main Outcome Measures:**

The presence of motor deficits was defined as a below-average standard score (≤40) on a comprehensive measure of motor skills, the Bruininks-Oseretsky Test of Motor Proficiency, second edition. HRQoL was evaluated using the Pediatric Quality of Life (PedsQL) Generic Core Scales.

**Results:**

Over 80% of childhood stroke survivors demonstrated below-average manual coordination, with over half of them showing deficits in both overall motor performance (62.5%) and fine manual control (54.2%). On the PedsQL, participants scored significantly lower on total (t=−2.9, *P*=.010, adjusted false discovery rate (FDR-adj.) *P*=.015) and psychosocial functioning (t=−3.4, *P*=.004, FDR-adj. *P*=.012). The motor domain significantly correlated with HRQoL: strength/agility showed the highest association with PedsQL total (*r*=.87, *P*<.001) and physical scores (*r*=.84, *P*=.002), whereas manual coordination correlated most strongly with psychosocial functioning (*r*=.88, *P*<.001).

**Conclusions:**

Motor impairments, especially in manual coordination and strength/agility, significantly affect quality of life after childhood stroke, warranting focused rehabilitation strategies.

Childhood stroke is a cerebrovascular event that occurs between 29 days and 18 years of age. Although childhood stroke may result from ischemic or hemorrhagic causes,^1^^,^^2^ both can lead to significant impacts on a child’s motor, cognitive, and psychosocial functioning, resulting in poor quality of life (QoL) and well-being of the affected children as well as documented increased parental stress.

Limited studies have assessed poststroke motor outcomes in children and adolescents using comprehensive motor assessment tools that evaluate specific components, such as fine motor precision, manual dexterity, bilateral coordination, balance, and strength, separately.^3^, ^4^, ^5^, ^6^ The detailed profiling of poststroke motor skills is important—it could enable rehabilitation specialists and allied health professionals to design targeted interventions to improve the overall motor outcomes.

Cooper et al^7^ adopted standardized motor assessments to evaluate motor outcomes in pediatric stroke survivors, focusing on fine and gross motor domains. Although Cooper’s study provided a characterization of neuromotor performance in pediatric stroke survivors, their cohort included neonatal stroke survivors in the analysis. Given the distinct differences in pathophysiological mechanisms between perinatal and childhood strokes,^8^^,^^9^ the motor performance and its impact on the overall functioning of childhood stroke survivors remained unclear.

Childhood stroke rehabilitation typically involves a multidisciplinary team, often with a primary focus. It is plausible that children with poorer motor outcomes may also be at increased risk for diminished mental well-being and reduced QoL. However, there is a lack of studies examining whether specific types or severities of motor impairment can predict broader functional and psychosocial outcomes. Understanding these associations could enable rehabilitation programs to tailor interventions more effectively across multiple domains, ultimately promoting better overall functioning and QoL in childhood stroke survivors.

Our study aims to address the research gap by conducting comprehensive and detailed profiling of motor performance, as well as exploring the associations between motor impairment, cognitive, psychosocial functioning, and health-related QoL in childhood stroke survivors.

## Methods

Ethics approval was obtained from the Institutional Review Board of the University of Hong Kong/Hospital Authority Hong Kong West Cluster (UW 24-090). Written consent was obtained from each participant.

### Study cohort

Children were considered for participation if they had a stroke diagnosis during the childhood period (between 29d and 18y old) and had prior admission to inpatient or outpatient settings at an acute hospital or a rehabilitation hospital for children.

Children with neonatal or perinatal strokes, coexisting brain injury caused by hypoxic ischemic or traumatic events, cerebral palsy, brain malignancy, genetic disorders primarily affecting the nervous system, and IQ<70 were excluded. For preterm infants (<37wk gestation), we excluded those with premature birth-associated strokes as noted in medical records. To investigate stroke-induced motor outcomes more accurately, children with intellectual disability were excluded in view of the association between motor ability and the extent of intellectual impairment,^10^ ensuring the attribution of motor outcomes to stroke.

Thirty-six children met the inclusion criteria. A total of 24 children with childhood stroke agreed to participate in this study; an occupational therapist (F.C.Y.C.) performed a comprehensive assessment on motor, cognitive, psychosocial, QoL, and functional performance for each participant in a research facility between March and August 2024.

### Neurologic and clinical demographics

#### Lesion

We reviewed neuroimaging data and reports to retrieve lesion location, laterality, and volume. Lesion location was categorized into “cortical,” “subcortical,” and “combined.” Lesion laterality was coded as left, right, and bilateral. Lesion volume was estimated using the ABC/2 technique,^11^, ^12^, ^13^ which is tested to be an accurate and reliable approach to measure primarily hemorrhage and infarct volume.

#### Age at assessment, age at stroke, time since stroke

Age at assessment, age of stroke, and time since stroke were calculated in a continuous manner. The calculation of age at assessment was based on the date of participants receiving their comprehensive motor, cognitive, and psychological assessments, whereas the calculation of age at stroke was based on the date of participants’ initial presentation of stroke symptoms. Time since stroke was the gap between these 2 time points.

#### Socioeconomic status

Educational qualifications and household income were used to indicate children’s socioeconomic status (SES).^14^ SES was estimated based on maternal education, paternal education, and household income scores. The educational status of both the mother and father was rated on a 3-point scale: (1) elementary school and below; (2) middle or high school; (3) college degree or above. Monthly household income (in HKD) was rescaled from a 5-point scale to a 3-point scale: (1) low income (< $29,999 HKD); (2) middle income ($30,000-$49,999 HKD); and (3) high income ($50,000 HKD and above).

### Measures

#### Motor outcome

The Bruininks-Oseretsky Test of Motor Proficiency, second edition (BOT-2) was used to evaluate motor performance in children and adolescents aged 4-21 years (supplemental table S1, available online only at http://www.archives-pmr.org/).^15^, ^16^, ^17^ BOT-2 measures performance in terms of (1) fine manual control; (2) manual coordination; (3) body coordination; and (4) strength and agility. Grip and pinch strength were used to evaluate upper limb strength.^18^^,^^19^ The Purdue Pegboard Test was used to measure dexterous hand function.^20^, ^21^, ^22^, ^23^, ^24^

#### Cognitive outcome

The Conners’ Continuous Performance Test third edition and Conners Kiddie Continuous Performance Test second edition were selected to assess attention and impulsivity in individuals aged 8 years and older and those aged 4-7 years, respectively.^25^, ^26^, ^27^, ^28^ Raven Progressive Matrices was used to evaluate and estimate general cognitive ability and nonverbal intelligence in children aged 6-18 years.^29^, ^30^, ^31^ The Behavior Rating Inventory of Executive Function, second edition was used to assess daily behaviors that reflect executive functioning in children and adolescents aged 5-18 years.^32^, ^33^, ^34^

#### Psychosocial functioning and QoL

The Strength and Difficulties Questionnaire parent-report and self-report were used to screen for emotional and behavioral problems in children and adolescents aged 4-17 years.^35^, ^36^, ^37^, ^38^, ^39^, ^40^ The Pediatric Quality of Life Inventory (PedsQL 4.0) Generic Core Scales and Family Impact Module measure the health-related quality of life (HRQoL) in children and adolescents aged 5-18 years.^41^, ^42^, ^43^ The Parental Stress Scale was used to assess the level of stress in the parents and investigates both the positive and negative facets of parenthood.^44^, ^45^, ^46^

#### Daily functioning

The Functional Independence Measure for Children (WeeFIM) was used to measure daily functional skills in children and adolescents aged 6 months to 21 years.^47^, ^48^, ^49^, ^50^, ^51^, ^52^, ^53^ Outcome evaluations were performed by a registered occupational therapist who had >4 years of relevant experience in pediatric neurologic rehabilitation.

### Statistical analysis

Clinical and neurologic demographic data were provided for the stroke cases. Continuous variables were expressed as the mean and standard deviation for normally distributed variables, and 95% CIs were provided for the mean. Categorical variables were presented as frequencies and percentages. To determine whether childhood stroke survivors would show worse performance than normative test samples across the motor, cognitive, and psychosocial domains, the cohort of children undertook a comprehensive assessment at 1 time point, and the assessment scores were compared with test-specific normative values or published normative data using 1-sample *t* tests. Effect sizes were calculated using Cohen’s d.^54^ Below-average or impairment rates will be reported only if the standardized assessment adopted includes specific cutoff scores. Correlations between motor outcomes, psychosocial and functional outcomes were calculated using Pearson’s partial correlation tests, controlling for type of stroke, time since stroke, stroke laterality, lesion location, lesion size, and SES. A correlation coefficient >0.7 indicates a strong correlation, whereas a moderate correlation falls between 0.5 and 0.7, and a coefficient <0.5 signifies a low correlation. Multiple comparisons were adjusted using the Benjamini–Hochberg method and were corrected using the false discovery rate approach with a false positive rate set at 0.05.^55^ Data analyses were conducted using IBM SPSS Statistics (version 29.0).^a^

## Results

### Characteristics of patients

We recruited 24 children and adolescents who fulfilled the inclusion and exclusion criteria from March to August 2024. The mean age at assessment was 13.3 years (SD 3.5y); mean age at stroke was 6.4 years (SD 5.8y); 9 (37.5%) were women; 11 (45.8%) and 13 (54.2%) suffered from arterial ischemic stroke and hemorrhagic stroke, respectively. All birth-related data were retrieved from the Clinical Management System of the Hospital Authority in Hong Kong; therefore, children and adolescents who were not born in hospitals under the HA may not possess birth records with information such as Apgar scores and birth weight. Table 1 included demographic data of the childhood stroke cohort.

**Table 1:** Patient demographic and stroke data.

Demographic and Stroke Characteristics	Total (*n*=24)
***Demographic data***	
**Sex, *n* (%)**	
Female	9 (37.5)
Male	15 (62.5)
**Mode of delivery, *n* (%)**	
Normal spontaneous delivery	14 (58.3)
Cesarean delivery	6 (25.0)
Missing information	4 (16.7)
**Gestational age, weeks, mean (SD)**	38.9 (1.3)
**Birth weight, g, mean (SD)**	3091 (207)
**1-min Apgar score, *n* (%)**	
8	6 (25.0)
9	6 (25.0)
10	3 (12.5)
Missing data	9 (37.5)
**5-min Apgar score, *n* (%)**	
9	5 (20.8)
10	10 (41.7)
Missing data	9 (37.5)
**Number of household members, *n* (%)**	
2	5 (20.8)
3	2 (8.3)
4	10 (41.7)
5	5 (20.8)
≥6	2 (8.4)
**Household size, ft^2^, *n* (%)**	
<400	9 (37.5)
400-699	7 (29.2)
700-999	4 (16.7)
≥1000	4 (16.7)
**Monthly household income, HKD, *n* (%)**	
<$12,000	4 (16.7)
$12,000-$29,999	5 (20.8)
$30,000-$49,999	6 (25)
$50,000-$79,999	4 (16.7)
≥$80,000	5 (20.8)
**Marital status, *n* (%)**	
Single	2 (8.3)
Married	19 (79.2)
Divorced	3 (12.5)
**Mother’s educational status, *n* (%)**	
Elementary school and below	2 (8.4)
Middle or high school	10 (41.6)
College degree or above	12 (50.0)
**Father’s educational status, *n* (%)**	
Elementary school and below	4 (16.6)
Middle or high school	8 (33.3)
College degree or above	12 (50.0)
**Mother’s occupational status, *n* (%)**	
Working	11 (45.8)
Unemployed	1 (4.2)
Housewife	12 (50.0)
**Father’s occupational status, *n* (%)**	
Working	20 (83.3)
Unemployed	3 (12.5)
Househusband	1 (4.2)
***Stroke characteristics***	
**Age at assessment, y, mean (SD)**	13.3 (3.5)
**Age at stroke, y, mean (SD)**	6.4 (5.8)
**Time since stroke, y, mean (SD)**	7.5 (4.3)
**Stroke type, *n* (%)**	
AIS	11 (45.8)
HS	13 (54.2)
**Lesion laterality, *n* (%)**	
Left	12 (50.0)
Right	9 (37.5)
Bilateral	3 (12.5)
**Lesion location, *n* (%)**	
Cortical	14 (58.3)
Subcortical	8 (33.3)
Combined	2 (8.3)
**Lesion volume, cm^3^, mean (SD)**	18.1 (49.1)

### Motor performance

#### Fine and gross motor composite

Compared with normative values, the overall mean of childhood stroke survivors was lower than the those without stroke on total motor composite (d=1.32), fine manual control (d=1.00), and manual coordination (d=1.56) (*P*<.001, adjusted false discovery rate (FDR-adj.) *P*<.001), and strength/agility (d=0.75, *P*=.001, FDR-adj. *P*=.001) (table 2). Body coordination scores were within normal range. Effect sizes are medium to large. Over 80% of children with childhood stroke displayed a below-average performance in manual coordination, and over half of the children had a below-average performance in overall motor performance and fine manual coordination (BOT-2 standard score ≤40).

**Table 2:** Motor performance compared to norm.

Motor performance	Mean (SD)	t	*P*	FDR-adj. *P*	Effect Size (*d*)	Below-Average Performance (%)
**BOT-2**						
TMC	39.25 (8.14)	−6.473	<.001	<.001	1.32	62.5
FMC	40.58 (9.46)	−4.877	<.001	<.001	1.00	54.2
MC	36.5 (8.65)	−7.648	<.001	<.001	1.56	83.3
BC	46.33 (12.53)	−1.434	.165	.165	0.29	37.5
S/A	42.33 (10.28)	−3.653	.001	.001	0.75	45.8
**Grip strength**						
Power grip (Dominant)	18.6 (8.85)	−3.518	.825	.825	1.02	/
Power grip (Nondominant)	13.7 (9.45)	−4.378	.159	.318	1.26	
**PPT**						
Dominant hand	13.13 (2.49)	−3.394	.002	.003	0.98	/
Nondominant hand	8.75 (5.3)	−4.752	<.001	<.001	1.37	
Both hands	7.76 (5.42)	−3.66	<.001	<.001	1.06	
Assembly	17.76 (11.5)	−5.581	.016	.016	1.61	

NOTE. Below-average performance in BOT-2 indicates a standard score ≤40.

Abbreviations: BC, Body Coordination; BOT-2, FMC, Fine Manual Control; MC, Manual Coordination; PPT, Purdue Pegboard Test; S/A, Strength and Agility; TMC, Total Motor Composite.

#### Grip strength

The grip strength of childhood stroke survivors was not significantly lower than the age-matched norm.^56^^,^^57^

#### Dexterity

The performance of childhood stroke survivors in all 4 domains: dominant hand (d=0.98, *P*=.002, FDR-adj. *P*=.003), nondominant hand (d=1.37, *P*<.001, FDR-adj. *P*<.001), both hands (d=1.06, *P*<.001, FDR-adj. *P*<.001), and assembly (d= 1.61, *P*=.016, FDR-adj. *P*=.016) was lower than the those without stroke.^22^ The effect sizes are large.

### Cognitive performance and executive functioning

Descriptive statistics for composite and subscale items are reported in supplemental table S2 (available online only at http://www.archives-pmr.org/). Nine participants did not complete the BRIEF self-report version because of the test-specific age limit (11-18y).

#### Attentional skills

Compared with norm-referenced scores of children without stroke (T score= 50),^26^, ^27^, ^28^ the mean scores of childhood stroke survivors in Hit Reaction Time (HRT) (d=.44, *P*=.041, FDR-adj. *P*=.123), HRT body coordination (d=.61, *P*=.007, FDR-adj. *P*=.063), and HRT SD + HRT Inter-Stimulus Interval Change (d=.45, *P*=.039, FDR-adj. *P*=.123) were higher than the those without stroke, although such differences were not significant after false discovery rate adjustment. Effect sizes are medium.

#### Nonverbal intelligence

Compared with norm-referenced scores in published data in a Chinese sample,^58^ mean scores of childhood stroke survivors were not significantly lower than the age-matched norm.

#### Parent-report EF

Compared with norm-referenced scores of children and adolescents with typical development (T score=50), childhood stroke survivors had significantly greater parent-reported executive dysfunction across several domains on the Behavior Rating Inventory of Executive Function, second edition scale, including Global Executive Composite (GEC), Cognitive Regulation Index (CRI), self-monitor, initiate, working memory, and task-monitor domains. For composite scores, there are indications of clinically elevated EF impairments in GEC (25.0%), Behaviour Regulation Index (BRI) (16.7%), Emotion Regulation Index (ERI) (20.8%), and CRI (25.0%). The self-monitor subscale had the highest clinically elevated rates of EF impairments (33.3%), followed by initiate (29.2%), working memory (25.0%), and task-monitor (25.0%). Effect sizes range from medium to large.

#### Self-report EF

Compared with normative values, childhood stroke survivors had significantly greater self-reported executive dysfunction across several domains, including GEC, ERI, CRI, shift, task completion, and working memory domains. For composite scores, results indicated impairments in EF across GEC (12.5%), BRI (8.3%), ERI (8.3%), and CRI (16.7%). Task completion and working memory subscales had the highest clinically elevated rates of EF impairments (20.8%), followed by shift (16.7%).

### Psychosocial functioning and Quality of Life

Table 3 provides a summary of psychosocial functioning performance and HRQoL compared to normative data. For the Strength and Difficulties Questionnaire parent-report, children with stroke demonstrated more emotional symptoms (d=.44, t=2.15, *P*=.043, FDR-adj. *P*=.080), peer problems (d=1.55, t=7.57, *P*<.001, FDR-adj. *P*<.001), and total difficulties (d=0.84, t=4.10, *P*<.001, FDR-adj. *P*<.001). No significant differences were found for conduct problems, hyperactivity, and prosocial behavior. Both peer problems and the overall difficulties scores had the highest impairment rate (83.3%). For the Strength and Difficulties Questionnaire self-report, these children obtained significantly higher scores in hyperactivity-inattention (d=1.27, t=4.58, *P*<.001, FDR-adj. *P*<.001), peer problems (d=0.65, t=2.36, *P*=.036, FDR-adj. *P*=.054), and total difficulties (d=0.73, t=2.64, *P*=.022, FDR-adj. *P*=.044). They also attained lower scores in prosocial behavior (d=−1.03, t=−3.71, *P*=.003, FDR-adj. *P*=.009).

**Table 3:** Psychosocial functioning and HRQoL compared to norm.

Psychosocial function	Mean (SD)	t	*P*	FDR-adj. *P*	Effect size (*d)*	Borderline impaired (%)	Impaired (%)
**SDQ-P**							
Emotional symptoms	2.83 (1.97)	2.15	.043	.080	0.44	8.3	16.7
Conduct problems	2.17 (1.43)	2.04	.053	.080	0.42	8.3	16.7
Hyperactivity	4.04 (1.2)	−0.07	.473	.568	−0.15	12.5	0.0
Peer problems	4.58 (1.21)	7.57	<.001	<.001	1.55	16.7	83.3
Prosocial behavior	7.38 (2.28)	0.51	.618	.618	0.10	8.3	37.5
Total difficulties	13.63 (3.76)	4.10	<.001	<.001	0.84	12.5	83.3
**SDQ-S**							
Emotional symptoms	3.62 (3.10)	1.53	.152	.182	0.43	7.7	38.5
Conduct problems	2.46 (2.37)	0.46	.654	.654	0.13	7.7	15.4
Hyperactivity	5.08 (1.38)	4.58	<.001	<.001	1.27	7.7	23.1
Peer problems	4.15 (1.99)	2.36	.036	.054	0.65	30.8	46.2
Prosocial behavior	5.46 (1.81)	−3.71	.003	.009	−1.03	7.7	61.5
Total difficulties	15.31 (6.43)	2.64	.022	.044	0.73	38.5	23.1
**PedsQL Generic Core Scales (proxy-report)**							
Physical health	73.05 (22.96)	−1.45	.167	.167	−0.36	/	
Psychosocial health	66.88 (16.03)	−3.42	.004	.012	−0.86		
Total score	69.23 (15.89)	−2.93	.010	.015	−0.73		
**PedsQL Generic Core Scales (self-report)**							
Physical health	81.25 (20.10)	0.22	.831	.969	0.05	/	
Psychosocial health	79.22 (15.94)	−0.04	.969	.969	−0.01		
Total score	79.92 (14.64)	0.09	.933	.969	0.02		
PedsQL Family Impact Module							
Parent HRQoL summary	76.20 (14.91)	2.23	.36	.108	0.46	/	
Family functioning summary	72.27 (20.24)	1.64	.115	.173	0.33		
Total	72.45 (15.07)	0.54	.596	.596	0.11		
**PSS (17-item)**	70.54 (7.71)	13.87	<.001	<.001	2.83	/	

NOTE. Borderline impaired is defined as scores meeting the “Borderline” criteria in SDQ-P and SDQ-S.

Impaired is defined as scores meeting the “Abnormal” criteria in SDQ-P and SDQ-S.

Abbreviations: PSS, Parental Stress Scale; SDQ-P, Strength and Difficulties Questionnaire parent-report; SDQ-S, Strength and Difficulties Questionnaire self-report.

For PedsQL Generic Core Scales, childhood stroke survivors in our sample on average had lower total scores (d=−.73, t=−2.93, *P*=.010, FDR-adj. *P*=.015) and psychosocial functioning scores (d=−.86, t=−3.42, *P*=.004, FDR-adj. *P*=.012) in the proxy-report version. Effect sizes were large. No significant difference was found in the self-report version. For the Family Impact Module, caregivers reported higher HRQoL scores (d=.46, t=2.23, *P*=.036, FDR-adj. *P*=.108) than the norm.

The cohort also demonstrated significantly higher scores on the Parental Stress Scale compared to nonclinical samples (d=2.83, t=13.87, *P*<.001, FDR-adj. *P*<.001).

### Functional outcomes

The raw scores and Developmental Functional Quotient (DFQ) of motor and cognitive domains and total WeeFIM ratings are presented in table 4 and supplemental table S3 (available online only at http://www.archives-pmr.org/).

**Table 4:** Functional performance compared to norm.

WeeFIM Domain	Mean (SD)	Mean DFQ (SD)	Good Functioning, *n* (%)	Moderate Functioning, *n* (%)	Poor Functioning, *n* (%)
Motor	87.29 (5.92)	95.29 (6.51)	95.83	4.17	0
Self-care	53 (4.57)	94.76 (7.93)	87.5	8.33	4.17
Mobility	34.29 (1.85)	97.98 (5.29)	91.67	8.33	0
Cognitive	27.88 (4.57)	79.64 (13.06)	37.5	45.83	16.67
Total	115.17 (7.59)	91.73 (5.72)	87.5	12.5	0

NOTE. Good functioning is defined as 85% of age-appropriate functioning; moderate functioning is defined as 70-84% of age-appropriate functioning; poor functioning is defined as <70% of age-appropriate functioning.

Abbreviation: DFQ, Developmental Functional Quotient.

The mean DFQ scores for the motor and total domains of childhood stroke survivors were slightly lower than those without stroke. Specifically, 95.8% and 87.5% of children achieved good functioning (at least 85% of age-expected performance) in the motor and total domains, respectively. However, < 40% of children attained good functioning in the cognitive domain, with most children (45.8%) falling into the moderate functioning category.

### Motor performance, Quality of Life, Function, and Psychosocial links

We performed partial correlation analyses between BOT-2 subdomains and WeeFIM motor DFQ and parent-report PedsQL generic module (table 5) adjusted for stroke type, time since stroke, stroke laterality, lesion location, lesion size, and SES. BOT-2 total motor composite, manual coordination, and strength/agility were found to have significant correlations with the 3 PedsQL summary scores. Strength/agility exhibited the strongest correlation with the PedsQL total score (*r*=.87, *P*<.001, FDR-adj. *P*=.005), followed by manual coordination (*r*=.86, *P*<.001, FDR-adj. *P*=.005), and total motor composite (*r*=.81, *P*=.004). Manual coordination showed the strongest correlation with the PedsQL psychosocial score (*r*=.88, *P*<.001, FDR-adj. *P*=.005), followed by strength/agility (*r*=.76, *P*=.011, FDR-adj. *P*=.028), and total motor composite (*r*=.74, *P*=.014, FDR-adj. *P*=.028). Strength/agility again showed the strongest correlation with the PedsQL physical score (*r*=.84, *P*=.002, FDR-adj. *P*=.008), followed by total motor composite (*r*=.74, *P*=.015, FDR-adj. *P*=.028). BOT-2 scores were not significantly associated with child-report PedsQL generic module. There were significant associations between motor DFQ and total motor composite (*r*=.73, *P*<.001, FDR-adj. *P*=.002), manual coordination (*r*= .56, *P*=.016, FDR-adj. *P*=.034), and strength/agility (*r*=.76, *P*<.001, FDR-adj. *P*=.002). There was no significant association between BOT-2 subdomains and psychosocial constructs (supplemental table S4, available online only at http://www.archives-pmr.org/).

**Table 5:** Association between motor performance, HRQoL and functional performance.

HRQoL/functional performance	PedsQL_Physical	0.37	0.66	0.62	0.84^⁎⁎^	0.74^⁎^
	PedsQL_Psychosocial	0.26	0.88^⁎⁎^	0.38	0.76^⁎^	0.74^⁎^
	PedsQL_Total	0.36	0.86^⁎⁎^	0.53	0.87^⁎⁎^	0.81^⁎^
	Motor DFQ	0.46	0.56^⁎^	0.49	0.76^⁎⁎^	0.73
	Cognitive DFQ	0.06	0.38	0.36	0.33	0.43
	Total DFQ	0.39	0.62^⁎^	0.64^⁎^	0.76^⁎⁎^	0.79^⁎⁎^
		BOT_FMC	BOT_MC	BOT_BC	BOT_S/A	BOT_TMC
	Motor outcomes					

Note 1:

PedsQL: Pediatric Quality of Life Inventory; FMC: Fine Manual Control; MC: Manual Coordination; BC: Body Coordination; S/A: Strength and Agility; TMC: Total Motor Composite.

Note 2:

1. Adjusted for stroke type, time since stroke, stroke laterality, lesion location, lesion size, SES.

2. Correlation with Significance (*p < 0.05, **p <0.01).

## Discussion

We demonstrated that childhood stroke survivors had significantly lower scores across all motor subscales compared with the typically developing population, and manual coordination appeared to be one of the most vulnerable motor areas after childhood stroke. Consistent with previous studies,^59^^,^^60^ our findings demonstrated significantly deteriorated HRQoL in our cohort compared to the normative population. More importantly, we further identified 2 key motor domains— manual coordination and strength/agility—that show the strongest associations with both the children’s and their caregivers’ QoL and daily functioning abilities.

Motor deficits are one of the most common sequelae after pediatric stroke.^7^^,^^61^, ^62^, ^63^ Given increased risks for motor dysfunction because of distinct pathophysiological mechanisms in neonatal/perinatal stroke survivors,^64^^,^^65^ our study focused exclusively on childhood stroke survivors for motor performance analysis. Three motor areas—fine manual control, manual coordination, and strength/agility—were found to be more impaired in these children. Impairments in manual coordination were particularly prevalent in our cohort. Manual coordination includes manual dexterity and upper limb coordination, whereas manual dexterity encompasses essential elements such as isolated finger movements and grip force control, and involves bimanual tasks with small items.^66^^,^^67^ Upper limb coordination is measured by arm and hand coordination quality.^16^ Stroke often leads to a decrease in muscle control contralaterally relative to the site of stroke in the brain^68^; as a result, bimanual coordination will be affected in childhood stroke survivors. The pronounced impairment in manual coordination can be explained by the following: (1) impaired inter-joint coordination in children with spastic hemiparesis^69^^,^^70^; (2) disruption of neural pathways that mediate bimanual coordination, affecting interlimb coupling^71^, ^72^, ^73^; (3) learned nonuse.^74^^,^^75^ Several plausible explanations may account for why fine manual control and strength/agility were comparatively less affected in our cohort. For fine manual control, motor and sensory deficits were observed in the unaffected limb. These deficits could be because of disruptions in neuronal networks.^76^^,^^77^ Despite this, childhood stroke survivors were trained to employ their stronger and more dexterous upper limb as the dominant side to cope with daily activities. For strength/agility—which primarily involves lower limbs and the trunk—we postulate that impairment severity is lower compared to the upper limb because of use-dependent mechanisms from forced use of the affected lower limb during walking.^78^^,^^79^

Our results revealed that children with poorer motor performance were associated with poorer QoL. We found that manual coordination and strength/agility played important roles in mediating the HRQoL of childhood stroke survivors. Consistent with the literature, O’Keeffe et al^80^ also found lower HRQoL in children with stroke. Existing studies on neonatal stroke established a relationship between manual ability and HRQoL.^81^ Our study not only confirmed the association between manual movements and HRQoL in childhood stroke survivors but also extended the findings by demonstrating a significant relationship between strength/agility and HRQoL. Existing studies on fundamental motor skills (FMS) in children may explain this phenomenon. FMS include stability, locomotor, and manipulative movements, such as dynamic balance, running and hopping, throwing, and kicking^82^^,^^83^; these skills functionally overlap with manual coordination and strength/agility. FMS are linked to psychosocial development and school performance; impaired manual coordination and strength/agility in children limit their play, social participation, and schooling, negatively affecting peer relationships, emotional well-being, and HRQoL. Beyond the established impacts on HRQoL, our findings reveal that impairments in manual coordination and strength/agility are associated with poor functional performance in childhood stroke survivors. These findings collectively highlight that manual coordination and strength/agility represent key motor domains impacting both HRQoL and functional outcomes, suggesting potential targets in pediatric neurorehabilitation.

### Implications for practice

Our study demonstrated that childhood stroke survivors experienced the greatest difficulties in manual coordination compared to other motor domains. Furthermore, children with impaired manual coordination and strength/agility reported a poorer functional capacity and QoL. Based on these findings, we emphasize the importance of implementing targeted interventions aimed at improving manual coordination and strength/agility. Such focused approaches are essential to enhancing functional independence and promoting overall well-being in childhood stroke survivors.

### Study limitations

Our study was limited by the small sample size. Although we recruited cases from the major pediatric rehabilitation children’s hospitals locally, future studies should involve other rehabilitation centers in Chinese Mainland. The study results should be interpreted with caution in view of the moderate degree of heterogeneity in terms of age at assessment, age at stroke, and time since stroke. Our study has adjusted these factors in statistical models. Although excluding children with intellectual disabilities enabled us to capture stroke-specific motor impairments, this method may underrepresent the comprehensive spectrum of functional impairments in childhood stroke survivors.

## Conclusions

Childhood stroke survivors demonstrated inferior motor performance compared to the typically developing population, particularly in manual coordination and strength/agility skills. These 2 motor areas are related to the HRQoL of affected children and their families, which warrants extra attention from clinicians to optimize neurorehabilitation goals.

## Supplier

a. SPSS, version 29.0; IBM.

## Disclosure

The investigators have no financial or nonfinancial disclosures to make in relation to this project.

## Appendix Supplementary materials

Image, application 1

Image, application 2

Image, application 3

Image, application 4

## Footnotes

Supplementary material associated with this article can be found, in the online version, at doi:10.1016/j.arrct.2025.100578.
