# Supplementary material for: Motor Function Profiling and Its Impact on Health-Related Quality of Life in Childhood Stroke Survivors
Source: Arch Rehabil Res Clin Transl. 2025 Dec 19;8(1):100578. doi: 10.1016/j.arrct.2025.100578 (PMC12988553; doi:10.1016/j.arrct.2025.100578)
Supplement: Supplementary file 1 [file mmc1.docx]

| **Outcome measure** | **Details** |
| --- | --- |
| **Motor** | |
| 1. Bruininks-Oseretsky Test of Motor Proficiency, Second Edition (BOT-2) | The BOT-2 is a comprehensive, age-normed, standardized motor assessment for children aged 4 to 21 years.^15^ This motor assessment is tested with satisfactory psychometric properties, including internal consistency, test-retest reliability, specificity and sensitivity.^16,17^ We adopted the complete form as a measurement tool, and it encompasses eight subtests which are categorized into four main areas: 1) Fine manual control, 2) Manual coordination, 3) Body coordination, and 4) Strength and agility. Successful administration of the BOT-2 complete form provides a total motor composite (TMC) standard score that measures the child’s motor performance across the four motor areas, and four composite scores in the four motor areas. Sex-specific norms were adopted instead of combined norms in view of the moderate effect of gender in specific subtests.^16^ |
| 1. Grip and pinch strength | They are crucial components in hand function assessment, serving as essential parameters for evaluating upper limb strength in relation to daily life functions across all age groups.^18,19^ In our study, we measured full grip, lateral, and tripod pinch using Jamar grip and pinch dynamometers. Grip strength is expressed in the unit of kilogram-force (kgf). |
| 1. Purdue Pegboard Test (PPT) | Dexterous hand function is frequently evaluated in stroke patients, and it refers to reaching, gripping, and lifting movements performed by the fingers.^20^ We measure dexterity using the Purdue Pegboard Test (PPT), a fine motor coordination test of satisfactory sensitivity.^21,22^ It can be applied to stroke survivors^23^ and has a norm for all ages over 5 years.^24^ The performance in four domains: dominant hand, non-dominant hand, both hands, and assembly, was recorded in our study. |
| **Cognition** | |
| 1. Conners' Continuous Performance Test 3rd Edition (CPT-3) and Conners Kiddie Continuous Performance Test 2nd Edition (K-CPT-2) | The appropriate assessment tool was selected depending on the child’s age at assessment. The Conners CPT-3 and Conners K-CPT-2 are computerized measures to assess attention and impulsivity in individuals aged 8 years and older and those aged 4 to 7 years, respectively.^25^ Higher T-scores generally indicate poorer performance, except for the variable Hit Reaction Time (HRT). Both abnormally high and low T-scores imply underlying problems. For all other variables except HRT, a T-score falling within the range of 55-59 will be counted towards the borderline impaired category, while a T-score above 59 will be counted towards the impaired category. However, previous studies have shown that specific composite indicators produce higher classification accuracy than individual indicators alone in CPT.^26,27^ We chose to use the sum of HRT Standard Deviation (SD) and HRT Inter-Stimulus Interval Change (ISIC) as the composite indicator in our study in view of its good classification accuracy and sensitivity.^28^ |
| 1. Raven Progressive Matrices (RPM) | To evaluate and estimate general cognitive ability and nonverbal intelligence.^29^ Participants are asked to complete the Standard Progressive Matrices (SPM) under the supervision of researchers. The SPM is a 60-item test designed for children and adolescents aged between 6 and 18 years; participants are instructed to select the correct figure from the six options presented to them.^30,31^ |
| 1. The Behavior Rating Inventory of Executive Function, 2^nd^ edition (BRIEF-2) | Assesses daily behaviors that reflect executive functioning (EF) in children and adolescents aged 5-18 years old.^32^ Both the BRIEF-2 parent and self-report forms were administered; the latter was administered to children or adolescents aged 11 to 18 years. There are four composite scores for both forms: Global Executive Composite (GEC), Behavioral Regulation Index (BRI), Emotional Regulation Index (ERI), and Cognitive Regulation Index (CRI). T scores were obtained for each scale in an age- and sex-specific manner, with higher T scores indicating more impaired EF.  Self-report measure of executive performance was chosen to capture participants’ EF abilities in daily functioning more comprehensively than performance-based measures.^33,34^ |
| **Psychosocial functioning and QoL** | |
| 1. The Strength and Difficulties Questionnaire parent-report and self-report (SDQ-P & SDQ-S) | SDQ-P is a screening questionnaire with good psychometric properties for emotional and behavioral problems in children and adolescents aged 4 to 17.^35-37^ SDQ-S is designed for children and adolescents aged 11-17 years to complete.^38^ It is a validated measure for screening emotional and behavioral problems in adolescents.^39^ The total difficulties score is generated by adding scores from all scales except the prosocial scale.^40^ The Chinese translations of both informant versions were adopted in our study. |
| 1. The Pediatric Quality of Life Inventory (PedsQL^TM^ 4.0) Generic Core Scales and Family Impact Module | PedsQL Generic Core Scales measure the health-related quality of life (HRQoL) in children and adolescents aged 5-18 years.^41^ This study adopted both the parent proxy-report and self-report versions, which evaluate the child’s perceptions of their health-related quality of life (HRQoL) as well as the caregiver’s perceptions of the child’s HRQoL.^42^ Three summary scores can be generated: a Total Quality of Life Score, a Physical Health Summary Score (physical functioning), and a Psychosocial Health Summary Score (emotional, social, school). PedsQL Family Impact Module is a parent-report measure indicating the impact of the child’s health on parent’s HRQoL and family functioning.^43^ For both generic and family impact versions, higher scores indicate enhanced functioning. |
| 1. Parental Stress Scale (PSS) | PSS is an 18-item scale assessing the level of stress in the parents and investigates both the positive and negative facets of parenthood.^44,45^ In the validated Chinese version, one of the items on the original scale “There is little or nothing I wouldn't do for my children if it was necessary” was excluded in view of its ambiguous implications and poor reliability. The Chinese version of the PSS with 17 items, demonstrating excellent reliability, validity, and internal consistency, was used in our study.^46^ |
| **Daily functioning** | |
| 1. Functional Independence Measure for Children (WeeFIM) | Measure participants’ daily functional skills across three domains: self-care, mobility, and cognition.^47^ WeeFIM is an 18-item scale and uses a 7-level ordinal scale to measure functional abilities in children and adolescents aged 6 months to 21 years, and it has proved to have good reliability and validity.^48-50^ Developmental functional quotients (DFQ) were used to evaluate functional outcomes instead of WeeFIM raw scores in view of the effects of age on functional scoring in children below 7 years.^51^ DFQ is calculated by dividing WeeFIM scores by age-adjusted norms, then multiplied by 100; a score nearing 100 suggests a child performs at a level more aligned with age-appropriate expectations^51,52^. DFQ of motor and cognitive domains, and total score were also further categorized into three categories: “good”, “moderate”, and “poor”, based on the percentage of age-expected functioning.^53^ WeeFIM was scored by both clinical observation and caregiver interviews. |

**Supplementary Material 1**. Outcome measures
