# Supplementary material for: Motor Function Profiling and Its Impact on Health-Related Quality of Life in Childhood Stroke Survivors
Source: Arch Rehabil Res Clin Transl. 2025 Dec 19;8(1):100578. doi: 10.1016/j.arrct.2025.100578 (PMC12988553; doi:10.1016/j.arrct.2025.100578)
Supplement: Supplementary file 2 [file mmc2.docx]

| **Supplementary Table 2.** Cognitive performance compared to norm | | | | | | | | |
| --- | --- | --- | --- | --- | --- | --- | --- | --- |
| Measure of cognitive function | Mean (SD) | t | | *p* | FDR adj. *p* | Effect size (*d*) | Borderline impaired (%) | Impaired (%) |
| **RPM** | 39.71 (11.67) | -0.996 | | 0.330 | 0.330 | -0.29 | / | / |
| **CPT-3/KCPT-2** |  | |  |  |  |  |  |  |
| d' | 51.50 (9.14) | 0.804 | | 0.430 | 0.553 | 0.16 | 16.7 | 20.8 |
| Omissions | 54.88 (13.95) | 1.712 | | 0.100 | 0.150 | 0.35 | 8.3 | 29.2 |
| Commissions | 49.83 (8.12) | -0.101 | | 0.921 | 0.921 | -0.02 | 12.5 | 12.5 |
| Perseverations | 50.33 (7.04) | 0.232 | | 0.819 | 0.921 | 0.05 | 12.5 | 12.5 |
| HRT | 55.54 (12.52) | 2.168 | | 0.041 | 0.123 | 0.44 | 8.3 | 45.8 |
| HRT SD | 55.58 (13.87) | 1.972 | | 0.061 | 0.139 | 0.40 | 8.3 | 33.3 |
| HRT BC | 56.83 (11.28) | 2.967 | | 0.007 | 0.063 | 0.61 | 12.5 | 37.5 |
| HRT ISIC | 56.17 (16.62) | 1.817 | | 0.082 | 0.148 | 0.37 | 12.5 | 33.3 |
| HRT SD + HRT ISIC | 111.75 (26.29) | 2.189 | | 0.039 | 0.123 | 0.45 | / | / |
| **BRIEF-2 (Parent-report)** |  | |  |  |  |  |  |  |
| *GEC* | 55.38 (11.17) | 2.357 | | 0.027 | 0.073 | 0.48 | 12.5 | 25 |
| *BRI* | 53.67 (9.85) | 1.825 | | 0.081 | 0.117 | 0.37 | 0 | 16.7 |
| Inhibit | 50.71 (8.93) | 0.389 | | 0.701 | 0.701 | 0.08 | 12.5 | 0 |
| Self-Monitor | 56.71 (11.68) | 2.814 | | 0.010 | 0.065 | 0.57 | 12.5 | 33.3 |
| *ERI* | 53.54 (10.85) | 1.599 | | 0.123 | 0.160 | 0.33 | 4.2 | 20.8 |
| Shift | 54.88 (11.98) | 1.993 | | 0.058 | 0.106 | 0.41 | 12.5 | 16.7 |
| Emotional Control | 52.08 (9.70) | 1.052 | | 0.304 | 0.359 | 0.22 | 4.2 | 20.8 |
| *CRI* | 55.5 (11.48) | 2.347 | | 0.028 | 0.073 | 0.48 | 4.2 | 25 |
| Initiate | 55.92 (13.13) | 2.207 | | 0.038 | 0.082 | 0.45 | 4.2 | 29.2 |
| Working Memory | 57.54 (11.84) | 3.122 | | 0.005 | 0.065 | 0.64 | 8.3 | 25 |
| Plan/Organize | 54.17 (10.53) | 1.939 | | 0.065 | 0.106 | 0.40 | 8.3 | 16.7 |
| Task-Monitor | 56.54 (12.21) | 2.624 | | 0.015 | 0.065 | 0.54 | 12.5 | 25 |
| Organization of Materials | 51.17 (8.84) | 0.647 | | 0.524 | 0.568 | 0.13 | 12.5 | 4.2 |
| **BRIEF-2 (Self-report)** |  | |  |  |  |  |  |  |
| GEC | 56.0 (8.74) | 2.658 | | 0.019 | 0.042 | 0.69 | 12.5 | 12.5 |
| BRI | 51.40 (9.36) | 0.58 | | 0.571 | 0.628 | 0.15 | 0 | 8.3 |
| Inhibit | 49.13 (8.41) | -0.399 | | 0.696 | 0.696 | -0.10 | 0 | 0 |
| Self-monitor | 55.0 (10.88) | 1.779 | | 0.097 | 0.152 | 0.46 | 8.3 | 12.5 |
| ERI | 54.93 (8.46) | 2.26 | | 0.040 | 0.073 | 0.58 | 8.3 | 8.3 |
| Shift | 57.60 (9.96) | 2.954 | | 0.010 | 0.033 | 0.76 | 12.5 | 16.7 |
| Emotional Control | 51.27 (7.56) | 0.649 | | 0.527 | 0.628 | 0.17 | 0 | 4.2 |
| CRI | 57.60 (9.72) | 3.03 | | 0.009 | 0.033 | 0.78 | 4.2 | 16.7 |
| Task Completion | 59.33 (11.02) | 3.279 | | 0.005 | 0.033 | 0.85 | 8.3 | 20.8 |
| Working Memory | 58.73 (11.71) | 2.889 | | 0.012 | 0.033 | 0.75 | 8.3 | 20.8 |
| Plan/Organize | 54.33 (10.24) | 1.638 | | 0.124 | 0.170 | 0.42 | 16.7 | 12.5 |
| Note:  Borderline impairment/impairment rates are not applicable to HRT SD + HRT ISIC score, as it is an aggregate score with no normative data available. | | | | | | | | |
