# Supplementary material for: Motor Function Profiling and Its Impact on Health-Related Quality of Life in Childhood Stroke Survivors
Source: Arch Rehabil Res Clin Transl. 2025 Dec 19;8(1):100578. doi: 10.1016/j.arrct.2025.100578 (PMC12988553; doi:10.1016/j.arrct.2025.100578)
Supplement: Supplementary file 3 [file mmc3.docx]

| **Supplementary Table 3.** Functional performance (subdomains) | | |
| --- | --- | --- |
| **WeeFIM domain** | **Mean** | **SD** |
| *Self-care* | 32.54 | 3.73 |
| Eating | 6.54 | 0.88 |
| Grooming | 6.75 | 0.68 |
| Bathing | 6.29 | 1.27 |
| Dressing - Upper | 6.29 | 1.16 |
| Dressing - Lower | 6.58 | 0.93 |
| *Sphincter control* | 20.46 | 1.22 |
| Toileting | 6.75 | 0.68 |
| Bladder management | 6.92 | 0.28 |
| Bowel management | 6.79 | 0.66 |
| *Transfers* | 20.79 | 0.72 |
| Chair | 6.92 | 0.28 |
| Toilet | 6.96 | 0.2 |
| Tub, shower | 6.92 | 0.28 |
| *Locomotion* | 13.5 | 1.18 |
| Walk | 6.79 | 0.59 |
| Stairs | 6.71 | 0.62 |
| *Communication* | 11.38 | 2.1 |
| Comprehension | 5.83 | 1.13 |
| Expression | 5.54 | 1.22 |
| *Social cognition* | 16.5 | 3.07 |
| Social interaction | 6.33 | 1.17 |
| Problem-solving | 4.96 | 1.33 |
| Memory | 5.08 | 1.56 |
