# Supplementary material for: Motor Function Profiling and Its Impact on Health-Related Quality of Life in Childhood Stroke Survivors
Source: Arch Rehabil Res Clin Transl. 2025 Dec 19;8(1):100578. doi: 10.1016/j.arrct.2025.100578 (PMC12988553; doi:10.1016/j.arrct.2025.100578)
Supplement: Supplementary file 4 [file mmc4.docx]

| **Supplementary Table 4.** Correlation between motor performance and psychosocial outcomes | | | | | |
| --- | --- | --- | --- | --- | --- |
| SDQ-P_Emotion | 0.13 | 0.29 | 0.34 | 0.03 | 0.28 |
| SDQ-P_Conduct | 0.01 | 0.31 | 0.36 | 0.22 | 0.31 |
| SDQ-P_Hyperactivity | -0.26 | 0.30 | 0.18 | 0.35 | 0.24 |
| SDQ-P_Peer | -0.45 | 0.04 | -0.48 | -0.27 | -0.33 |
| SDQ-P_Prosocial | -0.30 | 0.15 | -0.09 | 0.04 | -0.02 |
| SDQ-P_Total | -0.14 | 0.41 | 0.25 | 0.12 | 0.26 |
| FI_Parent HRQOL | -0.13 | 0.14 | -0.31 | -0.01 | -0.02 |
| FI_Family functioning | -0.27 | 0.45 | 0.03 | 0.34 | 0.23 |
| FI_Total | -0.21 | 0.30 | -0.16 | 0.11 | 0.09 |
| PSS | 0.09 | -0.12 | 0.02 | 0.21 | 0.25 |
|  | BOT_FMC | BOT_MC | BOT_BC | BOT_SA | BOT_TMC |
| Note 1:  SDQ-P: The Strength and Difficulties Questionnaire (Parent-report); FI: Family Impact; PSS: Parental Stress Scale; FMC: Fine Manual Control; MC: Manual Coordination; BC: Body Coordination; S/A: Strength and Agility; TMC: Total Motor Composite  Note 2:  1. Adjusted for stroke type, time since stroke, stroke laterality, lesion location, lesion size, SES  2. Correlation with Significance (*p < 0.05, **p <0.01) | | | | | |
